# Enhanced Heterogeneous Fenton Degradation of Organic Pollutants by CRC/Fe_3_O_4_ Catalyst at Neutral pH

**DOI:** 10.3389/fchem.2022.892424

**Published:** 2022-04-14

**Authors:** Chuan Wang, Rui Jiang, Jingxin Yang, Pingshan Wang

**Affiliations:** ^1^ A Key Laboratory for Water Quality and Conservation of the Pearl River Delta, Institute of Environmental Research at Greater Bay, Ministry of Education, Guangzhou University, Guangzhou, China; ^2^ Economic Development Bureau of Yongzhou Economic and Technological Development Zone, Yongzhou, China

**Keywords:** heterogeneous fenton, CRC@Fe3O4 catalyst, degradation, methylene blue, adsorption

## Abstract

Fe_3_O_4_-based heterogeneous Fenton catalysts have been widely employed for degrading organic pollutants, however it is challenging to use them in highly efficient and recyclable application in wastewater treatment. In this work, carboxylate-rich carbon (CRC)-modified Fe_3_O_4_ magnetic particles are prepared by the sol-gel self-combustion method, where CRC is obtained from the carbonization of sodium gluconate. The CRC/Fe_3_O_4_ catalyst exhibits high heterogeneous Fenton degradation performance. The complete 10 mg L^−1^ methylene blue (MB) removal is achieved in 180 min under conditions of 10 mM H_2_O_2_ and 1.00 g of L^−1^ CRC/Fe_3_O_4_ at neutral pH. After five cycles, the structure and morphology of CRC/Fe_3_O_4_ composites remained unchanged and the catalytic activity also remained unaltered. Moreover, phenol, benzoic acid (BA), sulfamethazine (SMT), and tetracycline (TC) were also degraded in the heterogeneous Fenton reaction using CRC/Fe_3_O_4_ as a catalyst. The strong coordinating ability of –COOH/ –COO^–^ functionalities of CRC formed strong bonds with Fe(II/III) ions on the surfaces of Fe_3_O_4_ particles, which was conducive to adsorption of organic matter on the surface of the catalyst and promoted the occurrence of heterogeneous Fenton reactions. It was found that CRC/Fe_3_O_4_ had higher removal rates for the adsorptive exclusions of pollutants, such as TC and MB, whereas there were lower removal rates for phenol, BA, and SMT. This work brings potential insights for development of a novel adsorption-enhanced heterogeneous Fenton reaction for wastewater treatment.

## 1 Introduction

Fenton oxidation technology is the most representative advanced oxidation process (AOP). The Fenton process has gained widespread acceptance for efficient degradation of recalcitrant organic contaminants (Pignatello et al., 2006; Wang and Wang, 2020; Coha et al., 2021; Zeng et al., 2021). However, the requirement of low pH and generation of huge amounts of iron sludge hinder widespread application. In order to avoid the drawbacks of the traditional Fenton reaction, iron oxide heterogeneous Fenton-like technology has been developed recently, in which iron oxide-catalyzed decomposition of H_2_O_2_ occurs to generate OH (He et al., 2016; Thomas et al., 2021). In fact, heterogeneous Fenton oxidation possesses inherent advantages, such as a wide pH application range, lower peroxide consumption, and recyclability *via* catalyst regeneration, over homogeneous Fenton oxidation (Lai et al., 2021; Xiea et al., 2021). The principal objectives behind developing heterogeneous Fenton oxidation technology are to prepare highly efficient, cheap, and easy to separate solid-phase catalysts.

Fe_3_O_4_ has been widely studied for its excellent magnetic separation performance (Wu et al., 2015; Xu et al., 2018; Adewunmi et al., 2020). Its magnetic properties allow for easy, fast, and inexpensive separation from the reaction medium. In addition, the potential of Fe_3_O_4_ derives from the higher ability for degradation of recalcitrant pollutants compared to the conventional iron-supported catalysts due to the presence of both Fe(II) and Fe(III) species (Munoz et al., 2015). However, Fe_3_O_4_ magnetic nanoparticles agglomerate easily because of their highly specific surface energy, resulting in uneven particle size (Liu et al., 2020). Therefore, the exposed Fe_3_O_4_ is easily oxidized, resulting in the reduction of magnetic properties (Yan et al., 2009). Again, at low pH, Fe_3_O_4_ magnetic nanoparticles are easily etched, affecting their morphology and properties, whereas Fe_3_O_4_ Fenton catalytic performance is inhibited when the pH of solution is weakly alkaline.

In order to overcome the above drawbacks, functionalization of nanoparticles is expected to be an effective alternative method (Zhu et al., 2018). Wrapping of inorganic materials, organic functional groups, such as –COOH, –NH_2_, and –SH, and biological macromolecules on the particle surface or superficial modification improve stability and functionalities of particles (Wulandari et al., 2018; Khatamian et al., 2019; Wang et al., 2021). The as-obtained multifunctional magnetic nanoparticles possess broader application prospects.

For synthesizing Fenton catalysis, Fe_3_O_4_ is first modified by humic acid (HA) to impart elevated photosensitivity, followed by modification with organic chelating agents to enhance the iron cycle and superficial compounding of the polymer to improve dispersion and stability. Klamerth et al. (Klamerth et al., 2011) pointed out that HA is a good choice for the improved optical Fenton system with a pH value of 6.5. Hua et al. (Hua et al., 2021) reported that the coating of catechol polymer on Fe_3_O_4_ enhanced the iron cycle and promoted the Fenton reaction. Again, Xue et al. (Xue et al., 2019) used electrospinning technology to prepare porous polycaprolactone composite nano Fe_3_O_4_ membrane synergy of the porous surface and embedded Fe_3_O_4_ nanoparticles to degrade methylene blue in the Fenton reaction. The surface modification of Fe_3_O_4_ improved its stability and catalytic efficiency, which is a potential research direction of Fenton heterogeneous catalysis.

Hence, in this work, CRC/Fe_3_O_4_ magnetic composites were synthesized by the sol-gel self-combustion method (Qu et al., 2016). The CRC/Fe_3_O_4_ samples were characterized and applied in the Fenton process to degrade MB. The effects of H_2_O_2_ and catalyst dosage, initial MB concentration, and pH value on the catalytic activity were investigated. The stability and recyclability of catalysts were also evaluated. The adsorption and heterogeneous Fenton degradation of phenol, benzoic acid (BA), sulfamethazine (SMT), tetracycline (TC), and MB were discussed to propose a possible adsorption/catalytic mechanism.

## 2 Experimental

### 2.1 Chemicals

All chemicals were of analytical grade and used without further purification. Ferric chloride (FeCl_3_·6H_2_O), sodium gluconate (C_6_H_11_NaO_7_), hydrogen peroxide (H_2_O_2_, 30 wt.%), methylene blue (C_16_H_18_ClN_3_S), tetracycline (C_22_H_24_N_2_O_8_), sulfamethazine (C_12_H_14_N_4_O_2_S), phenol (C₆H₆O), and benzoic acid (C_6_H_5_COOH) were purchased from Sigma-Aldrich (China). All solutions and suspensions were prepared by using deionized water.

### 2.2 Preparation and Characterization of Catalysts

Preparation of CRC/Fe_3_O_4_ magnetic composites by sol-gel self-combustion: A certain mass of sodium gluconate and FeCl_3_·6H_2_O were dissolved in 20 ml of distilled water, followed by the addition of 1.0 mol L^−1^ of NaOH solution to adjust the pH to 7. The as-obtained solution was stirred continuously at room temperature for 30 min, followed by solvent evaporation on a petri dish at 80°C for 1–2 h to obtain the gel. The as-obtained gel was then transferred to the crucible, covered, heated in a muffle furnace at 350°C for 2 h, and neutrally cooled to room temperature. Thereafter, the synthetic composite was ground and crushed, washed several times with distilled water and ethanol, separated by magnet, and dried at 60°C for 2 h.

The morphology and size distribution of CRC/Fe_3_O_4_ were observed by a scanning electron microscope (SEM, Pheonom ProX, Netherlands). The phase structures were determined by X-ray diffraction (XRD, PANalytical-PW3040/60, Netherlands). To verify the formation of CRC, surface chemistry was analyzed using a Fourier transform infrared (FTIR) spectrometer (Bruker TENSOR II Hyperion 2000, Germany). Thermal stabilities of CRC/Fe_3_O_4_ were assessed by a thermogravimetric analyzer (TGA, TGA/DSC PerkinElmer- TGA4000, US). The zeta potentials of the catalyst suspensions at different pH values were determined by an analyzer (Zetasizer, Malvern 3000).

### 2.3 Degradation Procedures

The degradation procedures were carried out in a 100 ml beaker shaken at a speed of 200 rpm. In a typical reaction, 50 ml of MB solution of a certain concentration was prepared by adding the specified amount of CRC/Fe_3_O_4_. The pH of reaction solution was adjusted to the required value by using 1.0 mol L^−1^ of H_2_SO_4_ or 1.0 mol L^−1^ of NaOH solutions. Degradation reactions were initiated by adding H_2_O_2_ to the suspension after attainment of the adsorption equilibrium. At pre-determined time intervals, 0.5 ml of sample suspension was withdrawn and the on-going reaction was quenched immediately by adding 30 μL of pure methanol. The solid particles were separated from the solution using an external magnet. The supernatant liquid was collected for analysis. Each experiment was run in triplicate and the arithmetic mean of the three measured values was used in the reported data.

### 2.4 Analytical Methods

The concentration of MB was measured by UV-*vis* spectroscopy at the fixed wavelength of 660 nm, which is the maximum absorption wavelength of MB. The concentration of other organic matters (Phenol, BA, SMT, TC) were determined by liquid chromatography-mass spectrometry (H-class/ QDA, waters, United States). The total leached iron was measured using the orthophenantroline complexometric method (*λ* = 510 nm).

## 3 Results and Discussion

### 3.1 Formation of CRC/Fe_3_O_4_

**FIGURE 1:**
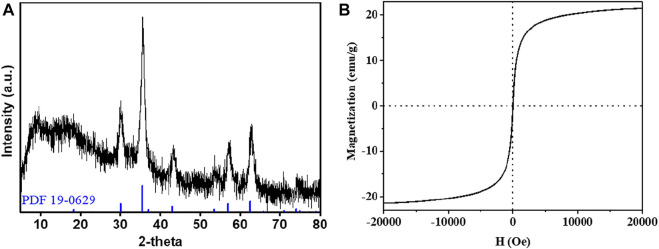
**(A)** XRD pattern and **(B)** magnetization curves of CRC/Fe_3_O_4_.

The XRD patterns of CRC/Fe_3_O_4_ contained sharp crystalline peaks at 2*θ* = 30.3, 35.6, 43.3, 53.7, 57.2, and 62.7° (Figure 1A), attributed to (220), (311), (400), (422), (511), and (440) planes of the cubic spinel structure of Fe_3_O_4_ (JCPDS 19–0629), respectively. In CRC/ Fe_3_O_4_, the absence of the characteristic peaks of carbon either individually or overlapped with the strong peaks of Fe_3_O_4_ inferred the presence of amorphous carbon. The magnetic properties of CRC/Fe_3_O_4_ were studied using a superconducting quantum interference device (SQUID) magnetometer at room temperature. The hysteresis loop of CRC/Fe_3_O_4_ (Figure 1B) indicated the magnetic saturation (Ms) of CRC/Fe_3_O_4_ was approximately 21.4 emu g^−1^.

**FIGURE 2:**
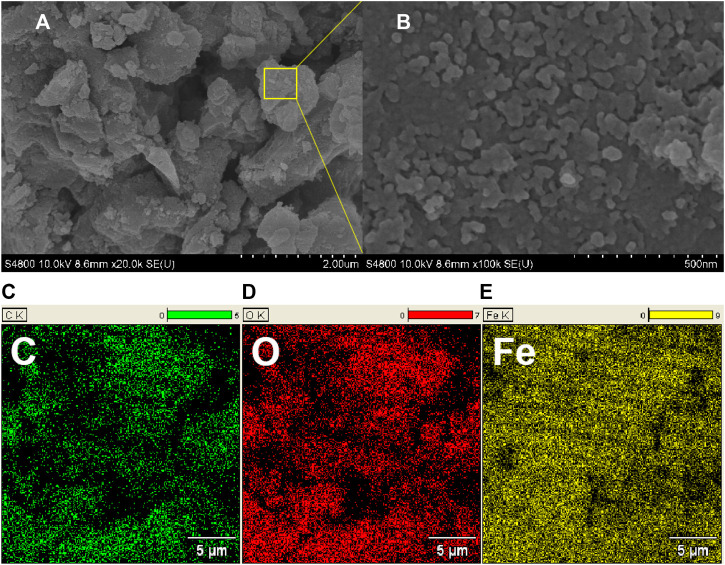
**(A,B)** SEM photomicrographs and **(C–E)** EDX mapping of C/ O/ Fe in CRC/Fe_3_O_4_.

From the SEM photomicrograph of CRC/ Fe_3_O_4_ (Figure 2), particles of uniform sizes and shapes were observed. The particle size distribution of cubic Fe_3_O_4_ was within 30–40 nm. Meanwhile, the energy dispersive X-ray (EDX) spectroscopy study verified the existence and even distribution of Fe, O, and C in CRC/ Fe_3_O_4_.

**FIGURE 3:**
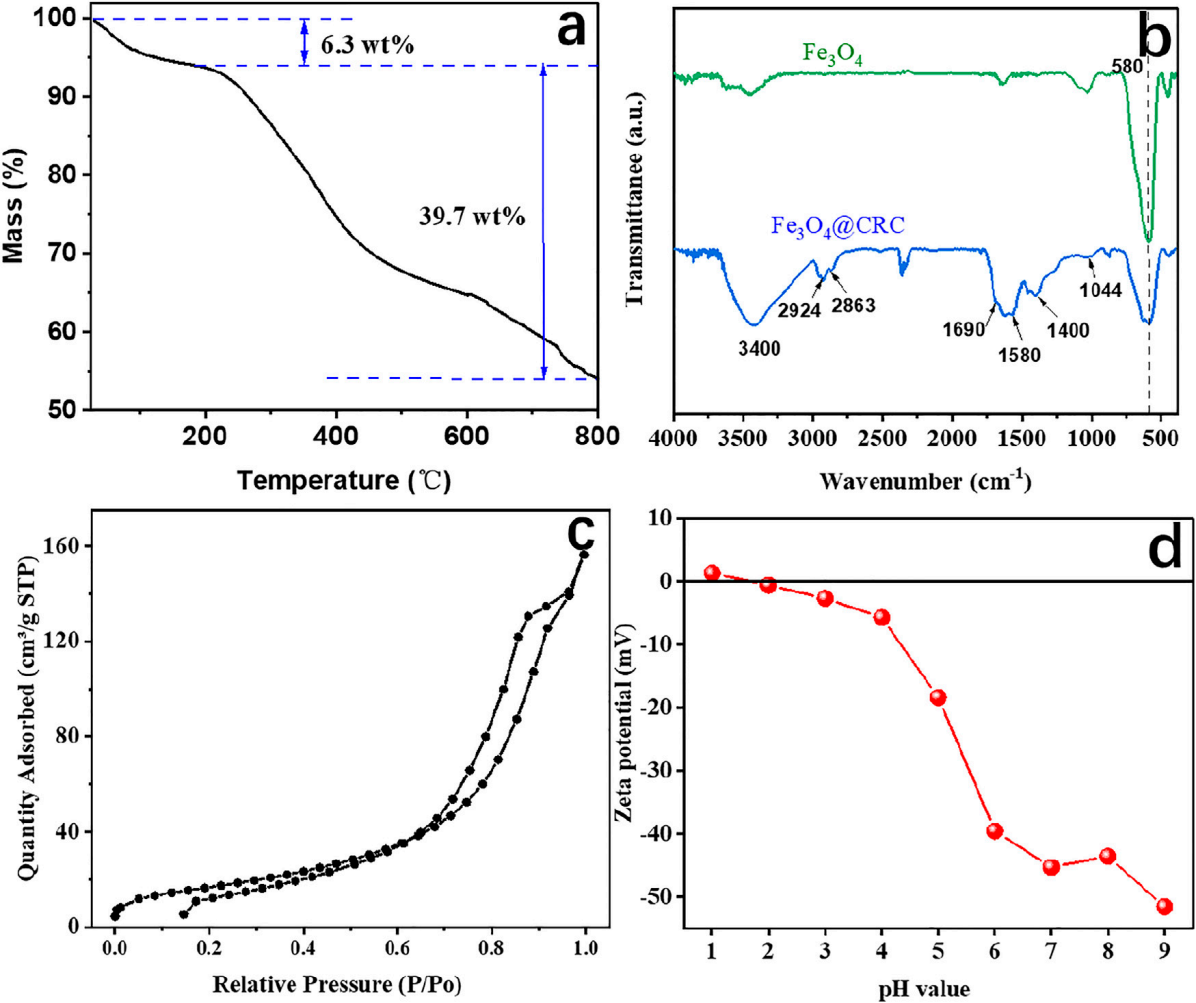
**(A)** TG curve, **(B)** FTIR spectra, **(C)** nitrogen adsorption/ desorption isotherms, and **(D)** zeta potential of CRC/Fe_3_O_4_.

The carbon content in CRC/Fe_3_O_4_ was determined by TG analysis performed under air (Figure 3A). The 6.3 wt% mass loss in CRC/Fe_3_O_4_ up to 190 °C was attributed to the loss of loosely adhered water and structural water molecules. However, major degradation took place within 190–800°C because of the combustion of carbon giving rise to a large weight loss of about 39.7 wt%. While considering that Fe_3_O_4_ would be converted to Fe_2_O_3_ when heated in air, the actual carbon content in CRC/ Fe_3_O_4_ could be estimated to be lower.

Figure 3B displays the FTIR spectra of the as-prepared samples. In Fe_3_O_4_, the presence of Fe–O was identified by the peaks around 580 cm^−1^ (Leng et al., 2013). In CRC/Fe_3_O_4_, the incorporation of CRC was envisaged from the prevalent CRC-specific functionalities, such as C–O, C–C, C=O, C–H, and O–H. For instance, the small peaks at 1,044 and 1,580 cm^−1^ indicated C–O *str.* and C–C *str.* of the aromatic ring, respectively (Qu et al., 2013). Again, peaks at 1,690 and 1,400 cm^−1^ were assigned to C=O *str.*, which envisaged the abundance of carboxylate functionalities in the carbon shell. The absorption peaks of H–C–H *str.* were detected at 2,924 and 2,863 cm^−1^ (Le et al., 2021). The peaks at 3400 cm^−1^ indicated the presence of large numbers of hydroxyl groups in CRC/ Fe_3_O_4_ (Kong et al., 2002).

Surface area, pore volume, and pore size distribution of CRC/Fe_3_O_4_ were analyzed by nitrogen adsorption-desorption techniques. According to Figure 3C, the type-IV isotherm pattern of CRC/Fe_3_O_4_ was characteristic of mesoporous materials. However, in CRC/Fe_3_O_4_, the prevalent narrower hysteresis loop and steep increase in adsorption at P/P_0_ close to 0.7 indicated the presence of macropores. The BET surface area and total pore volume were measured to be 42.44 m^2^ g^−1^ and 0.0125 cm^3^ g^−1^, respectively. Because of the significant population of carboxylate functionalities in CRC, the zero point charge of CRC/Fe_3_O_4_ was found to be approximately 1.7 (Figure 3D). Moreover, zeta potentials of CRC/Fe_3_O_4_ became more negative with the increasing suspension pH. Abundant numbers of negative charges on the surface of CRC/Fe_3_O_4_ benefited the sorption of positively charged metal ions, such as Fe(II) ions.

### 3.2 Heterogeneous Fenton Catalytic Activity of CRC/Fe_3_O_4_

#### 3.2.1 CRC/ Fe_3_O_4_ Catalytic Activity

**FIGURE 4:**
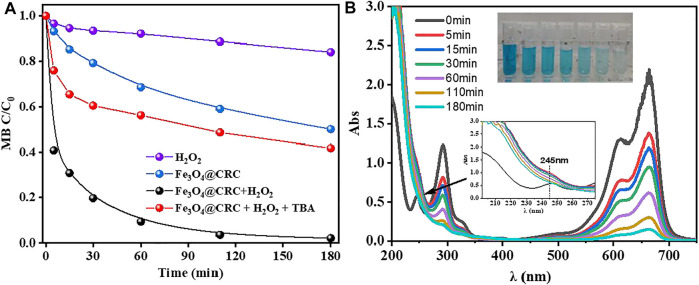
**(A)** Removal of MB under different conditions, **(B)** time-dependent UV–vis spectra for degradation of MB by CRC/Fe_3_O_4_ + H_2_O_2_ [(CRC/Fe_3_O_4_) = 1.00 g L^−1^, (MB) = 10 mg L^−1^, (H_2_O_2_) = 10 mM, (TBA) = 100 mM, and pH_0_ = 7].

The catalytic activity of CRC/Fe_3_O_4_ was evaluated by MB degradation experiments through the Fenton reaction. As indicated in Figure 4A, after 180 min of reaction, MB was removed almost completely, whereas in absence of the catalyst, the degradation of MB in the H_2_O_2_ system was kinetically slower to ensure only 16% removal at 180 min. Again, in the absence of H_2_O_2_, 50% of MB was adsorbed onto CRC/Fe_3_O_4_. Interestingly, in Figure 4B, absorption peaks at 660/ 290 and 245 nm decreased significantly and disappeared, respectively, after initiation of the reaction. At the same time, the UV absorption within 200–270 nm increased significantly, indicating degradation of MB and the simultaneous formation of some small molecular organics.

TBA was added into the CRC/Fe_3_O_4_-H_2_O_2_ system to capture ·OH, as shown in Figure 4A. Herein, after TBA was added, the removal rate of MB dropped to 59% at 180 min, confirming that ·OH is the most important active oxygen species in the system. At neutral conditions, there were few iron ions dissolved in the bulk solution, and the reaction mainly occurred on the surface of the catalyst. The CRC/Fe_3_O_4_ catalytic degradation of MB can be attributed to the synergistic effects of catalyst adsorption and degradation. Since the surface of the sodium gluconate-modified Fe_3_O_4_ catalyst possessed a highly developed pore structure and plenty of functionalities, the porous CRC/Fe_3_O_4_ structure provided a larger specific surface area to adsorb a higher amount of MB, thereby increasing the relative concentration of pollutants. At the same time, H_2_O_2_ also reacted on the surface of the catalyst to form ·OH and reacted with MB. Adsorption is a controlled step of MB degradation.

#### 3.2.2 Effects of Degradation Conditions

**FIGURE 5:**
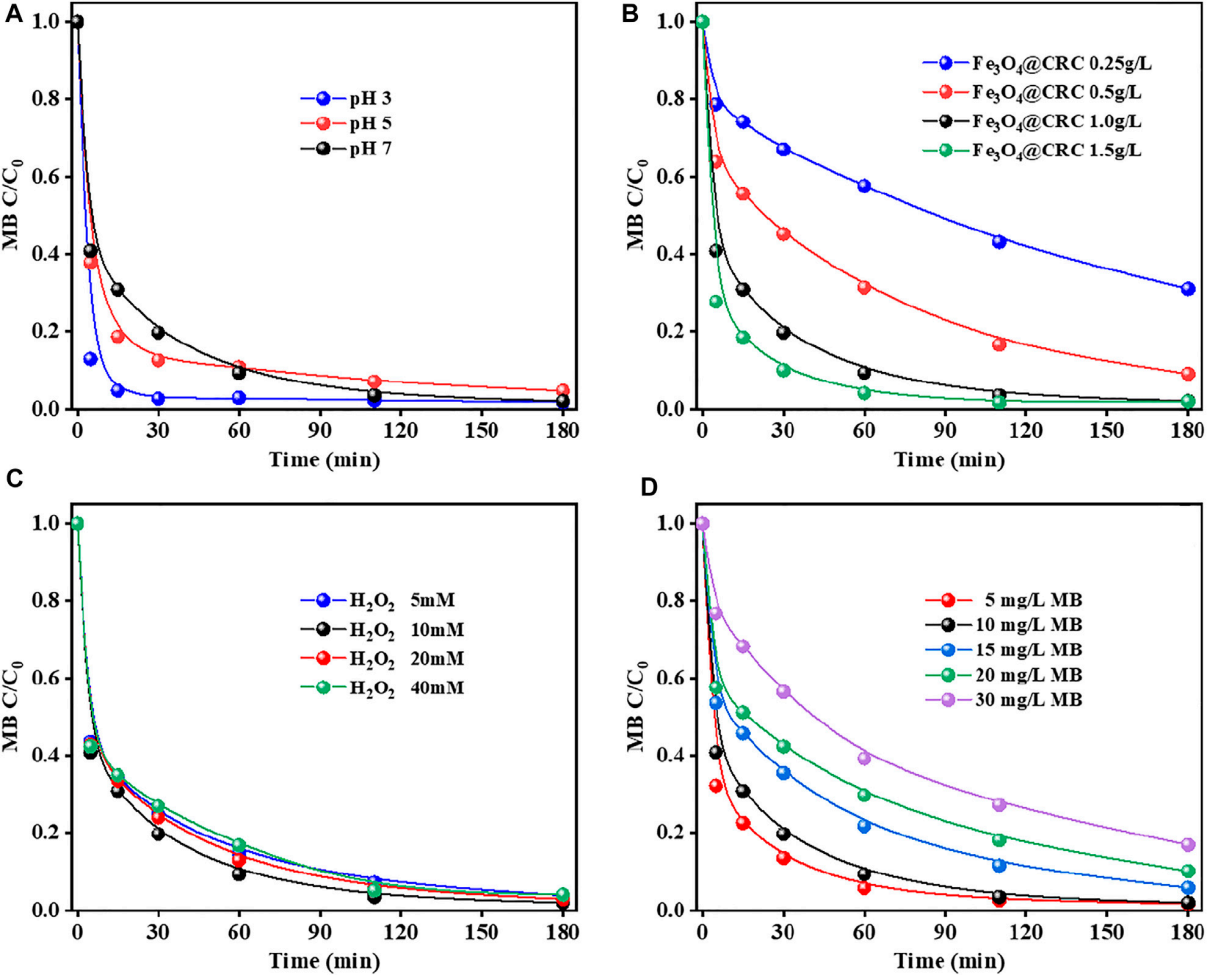
Factors affecting MB degradation by CRC/Fe_3_O_4_: **(A)** initial solution pH, **(B)** catalyst dosage, **(C)** H_2_O_2_ concentration, and **(D)** MB concentration [(CRC/Fe_3_O_4_) = 1.00 g L^−1^, (MB) = 10 mg L^−1^, (H_2_O_2_) = 10 mM, and pH_0_ = 7].

In the study of conventional Fenton treatment, the best result can be obtained at pH 3. Under neutral or alkaline conditions, it is not conducive to treat MB wastewater because of the restricted degradation treatment and research of MB. Herein, the catalytic activities of CRC/Fe_3_O_4_ were studied at different pH values. As can be seen from Figure 5A, MB could be completely degraded within 180 min within pH 3-7, indicating that CRC/ Fe_3_O_4_ has good applicability in a wide pH range. In fact, the fastest degradation rate at pH 3 was mainly caused by the dissolution of iron ions (∼0.16 mmol/L), replicating the homogeneous Fenton reaction. However, no significant difference in the reaction rates were observed for pH = 5 and 7 because of the poor iron ion concentration (<0.01 mmol/L). The heterogeneous Fenton catalytic degradation reaction occurred after adsorption of MB on the catalyst.

Figure 5B shows the degradation of MB by CRC/Fe_3_O_4_ under different catalyst dosages. Results showed that when the dose of CRC/Fe_3_O_4_ increased from 0.25 to 1.00 g L^−1^, the removal efficiency of MB improved gradually, while the removal efficiency exhibited negligible change when the dose of CRC/Fe_3_O_4_ increased beyond 1.50 g L^−1^. This was because with the increase in the amount of catalyst, the surface area and active sites, responsible for the acceleration of H_2_O_2_ decomposition, also increased. However, with the further increment in the amount of catalyst, agglomeration of catalyst particles and excessive iron accumulation removed **·**OH, which inhibited the increment of the removal efficiency of MB (Phan et al., 2018; Qin et al., 2018; Xiao et al., 2018). When the amount of catalyst was varied within 1.00–1.50 g L^−1^, no significant effect on the final removal rate of MB was observed.

The concentration of H_2_O_2_ plays an important role in the heterogeneous Fenton process because it is directly related to the amount of **·**OH produced. Generally, the amount of **·**OH produced is directly proportional to the concentration of H_2_O_2_, that is, the higher the concentration of H_2_O_2_, the more **·**OH produced, and the more degradation of pollutants. However, when the concentration of H_2_O_2_ exceeds the critical value, the degradation rate may be limited because of the reaction of **·**OH with excess H_2_O_2_ and the conversion into a hydrogen peroxide radical (Liu et al., 2019). As shown in Figure 5C, with an increase in the initial H_2_O_2_ concentration from 5 to 20 mM, the removal rate of MB did not improve significantly. This observation inferred that a lower concentration of H_2_O_2_ is sufficient for the degradation of MB, and CRC/Fe_3_O_4_ as a catalyst has a good utilization rate of peroxide.

The initial concentration of MB affects the adsorption and degradation of the catalyst. As seen in Figure 5C, with the increase in MB concentration, the degradation efficiency was found to decrease gradually. When the initial MB concentration was 5 mg L^−1^, 90% MB removal could be achieved after 30 min. The degradation of 10 mg L^−1^ MB was similar to that of 5 mg L^−1^. However, the degradation rates of MB at 15, 20, and 30 mg L^−1^ were 46.41, 42.47, and 23.30%, respectively. However, the degradation rates of MB at 15, 20, and 30 mg L^−1^ were 46.41, 42.47, and 23.30%, respectively (Figure 5D). The higher the MB concentration, the longer the duration of the degradation process. This was because the amount of **·**OH produced in the reaction system became constant when the dose of the CRC/Fe_3_O_4_ catalyst and H_2_O_2_ were constant. For the lower dye concentration, the **·**OH in the solution would be relatively excessive. However, with the increase in dye concentration, the **·**OH produced in the solution should be relatively insufficient. Therefore, it became necessary to prolong the reaction time to remove the higher concentration of the MB solution.

#### 3.2.3 CRC/Fe_3_O_4_ Stability and Reusability

**FIGURE 6:**
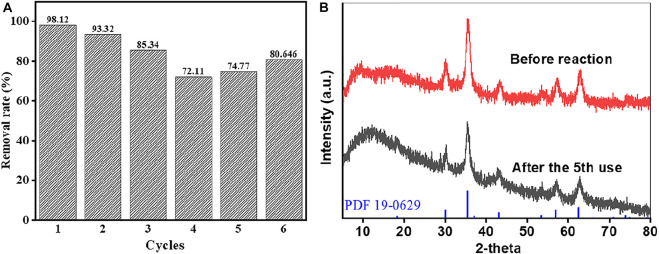
Reusability of CRC/Fe_3_O_4_ for the degradation of MB: **(A)** removal rates for the six consecutive cycles and **(B)** XRD patterns before reaction/after five consecutive cycles [(CRC/Fe_3_O_4_) = 1.00 g L^−1^, (pollutant) = 10 mg L^−1^, (H_2_O_2_) = 10 mM, and pH_0_ = 7].

Stability and reusability of materials are extremely important for an effective catalyst. In this study, MB was continuously degraded by the heterogeneous Fenton reaction with CRC/Fe_3_O_4_ under the same conditions, and its stability was evaluated. As shown in Figure 6A, the catalytic performance of CRC/Fe_3_O_4_ declined in six consecutive cycles, because of the possible loss of active sites and adsorption of by-products during MB degradation (Doan et al., 2019). Importantly, after five continuous uses, the CRC/Fe_3_O_4_ composite still maintained high degradation efficiency. Additionally, at pH = 7, the total dissolved concentration of leached iron was lower than that of the detection limit. As demonstrated in Figure 6B, there was no obvious change in XRD pattern after five consecutive cycles.

**FIGURE 7:**
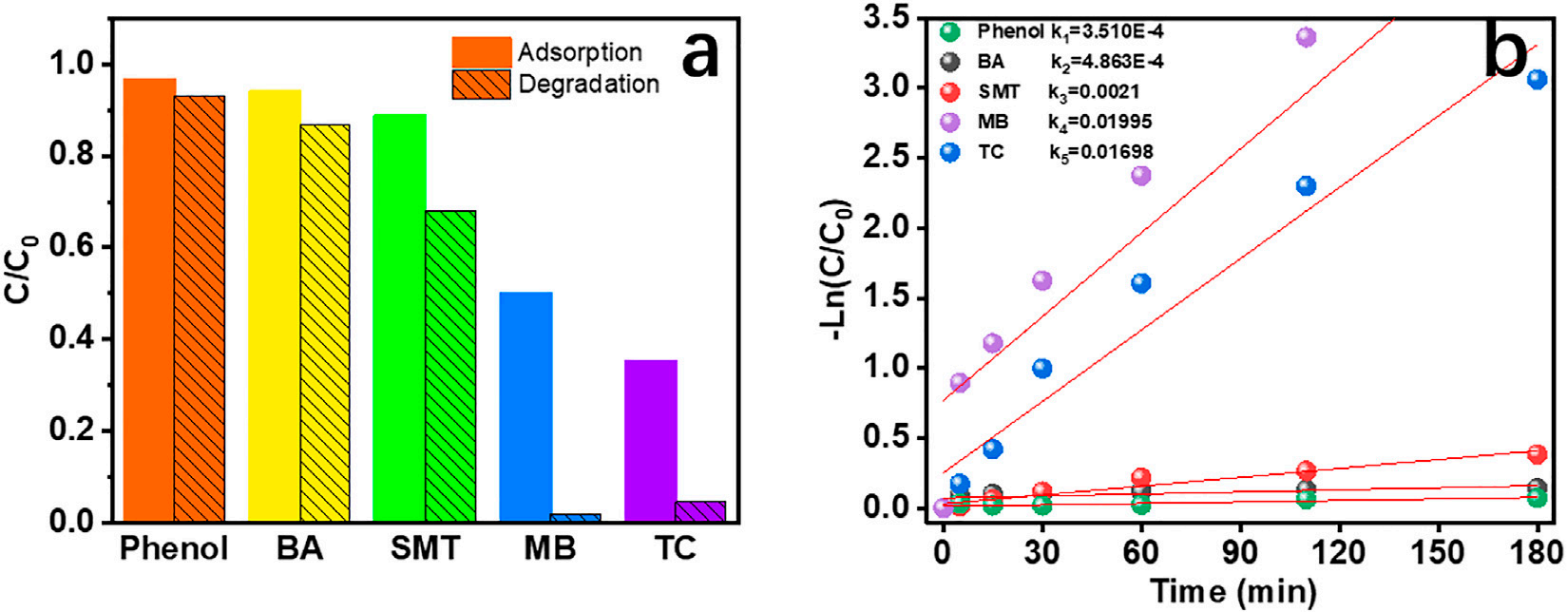
**(A)** Adsorption and degradation efficiency and **(B)** degradation rate of different pollutants by the CRC/Fe_3_O_4_ heterogeneous Fenton system in 180 min [(CRC/Fe_3_O_4_) = 1.00 g L^−1^, (pollutant) = 10 mg L^−1^, (H_2_O_2_) = 40 mM, and pH = 7].

Figure 7A shows the adsorption and degradation efficiency of CRC/Fe_3_O_4_ for phenol, BA, SMT, MB, and TC, of which adsorption and degradation of phenol and BA were poor. The adsorption/removal rates of SMT, MB, and TC were 12/20%, 50/98%, and 65/95%, respectively. The degradation trend of organic pollutants was similar to the adsorption trend on the surface of the CRC/Fe_3_O_4_ catalyst, since adsorption of contaminants promotes their catalytic degradation. The same trend is also reflected in the degradation rate in Figure 7B. The synergistic effects of adsorption and catalytic degradation significantly improved the removal effect of organic matter.

## 4 Conclusion

This study reports the spontaneous synthesis of CRC/Fe_3_O_4_ magnetic particles by the sol-gel combustion method and their catalytic properties and related mechanisms. CRC is conducive towards adsorption of organic matter on the surface of the catalyst and promotes the occurrence of heterogeneous Fenton reactions. Under neutral conditions, the CRC/Fe_3_O_4_ heterogeneous Fenton reaction predominantly occurs on the surface of the catalyst. Therefore, adsorption of pollutants on the surface of the catalyst is closely related to the CRC/Fe_3_O_4_ heterogeneous Fenton degradation effect, and the larger the adsorption amount, the higher the degradation removal rate. Experimental results infer that CRC/Fe_3_O_4_ is negatively charged and therefore possesses better adsorption and degradation of positively charged substances. Further modification of materials is expected to enable selective removal of specific contaminants.

## Data Availability

The original contributions presented in the study are included in the article/Supplementary Material, further inquiries can be directed to the corresponding authors.
