# Corrigendum: Enhanced Heterogeneous Fenton Degradation of Organic Pollutants by CRC/Fe_3_O_4_ Catalyst at Neutral pH

**DOI:** 10.3389/fchem.2022.939407

**Published:** 2022-06-14

**Authors:** Chuan Wang, Rui Jiang, Jingxin Yang, Pingshan Wang

**Affiliations:** ^1^ A Key Laboratory for Water Quality and Conservation of the Pearl River Delta, Institute of Environmental Research at Greater Bay, Ministry of Education, Guangzhou University, Guangzhou, China; ^2^ Economic Development Bureau of Yongzhou Economic and Technological Development Zone, Yongzhou, China

**Keywords:** heterogeneous fenton, CRC/Fe_3_O_4_, degradation, methylene blue, adsorption

In the original article, two author names were incorrectly spelled as “Jinxing Yang and Pinshan Wang.” The correct spellings are “Jingxin Yang and Pingshan Wang.”

The authors apologize for this error and state that this does not change the scientific conclusions of the article in any way. The original article has been updated.

